# Tuberculous pericarditis

**DOI:** 10.7705/biomedica.4911

**Published:** 2020-08-20

**Authors:** Leonardo F. Jurado, Bibiana Pinzón, Zandra R. de la Rosa, Marcela Mejía, Diana M. Palacios

**Affiliations:** 1 Departamento de Patología y Laboratorios, Hospital Universitario Fundación Santa Fe de Bogotá, Bogotá, D.C., Colombia Departamento de Patología y Laboratorios Hospital Universitario Fundación Santa Fe de Bogotá BogotáD.C Colombia; 2 Departamento de Patología, Facultad de Medicina, Universidad Nacional de Colombia, Bogotá, D.C., Colombia Universidad Nacional de Colombia Departamento de Patología Facultad de Medicina Universidad Nacional de Colombia BogotáD.C Colombia; 3 Departamento de Microbiología, Facultad de Medicina, Universidad Nacional de Colombia, Bogotá, D.C., Colombia Universidad Nacional de Colombia Departamento de Microbiología Facultad de Medicina Universidad Nacional de Colombia BogotáD.C Colombia; 4 Departamento de Imágenes Diagnósticas, Hospital Universitario Fundación Santa Fe de Bogotá, Bogotá, D.C., Colombia Departamento de Imágenes Diagnósticas Hospital Universitario Fundación Santa Fe de Bogotá BogotáD.C Colombia

Tuberculous pericarditis is an infrequent but serious form of tuberculosis. Its diagnosis is difficult and often delayed or not even reached, which results in complications such as constrictive pericarditis with high mortality rates ^1^. In 2017, there were 10 million cases of active tuberculosis worldwide and 1.3 million related deaths making tuberculosis the leading cause of death by a single pathogen worldwide ^2^. In Colombia, 13,626 new cases of tuberculosis were reported during 2016 of which 83% (11,338 cases) corresponded to pulmonary tuberculosis and 17% (2,288 cases) to extrapulmonary tuberculosis while 37 cases (1.6%) of these corresponded to tuberculous pericarditis ^3^.

We describe here the case of tuberculous pericarditis in a man with no apparent risk factors to develop the disease, which reinforces the concept that no predisposing condition is necessary to develop tuberculosis ^4^.

A 62-year-old man presented to the emergency room with a history of malaise, fever, cough, dyspnea, and loss of 5 kg of weight in the previous 30 days. His initial assessment showed normal vital signs and no abnormalities in the white blood cell count; the erythrocyte sedimentation rate was 56 mm/h, the C-reactive protein was 10.66 mg/l, and the procalcitonin level was less than 0.5 ng/ml; the serology for HIV was negative.

**Figure 1:**
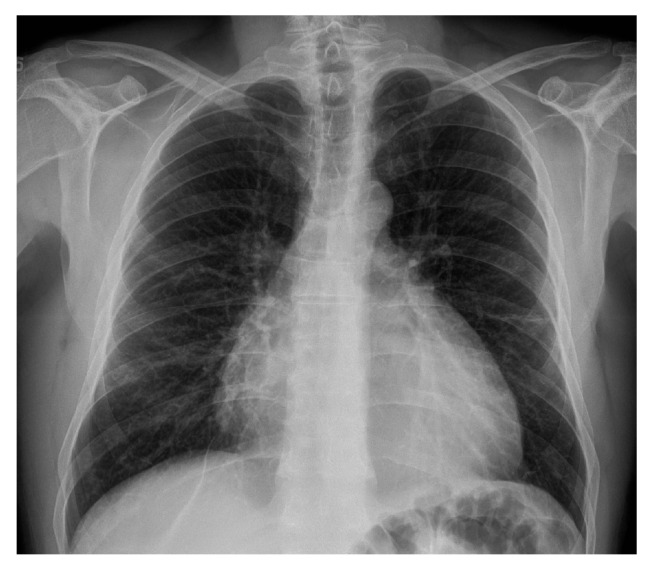
Chest X-ray. PA projection. Global cardiomegaly with rounded cardiac shape. No opacities were observed.

**Figure 2:**
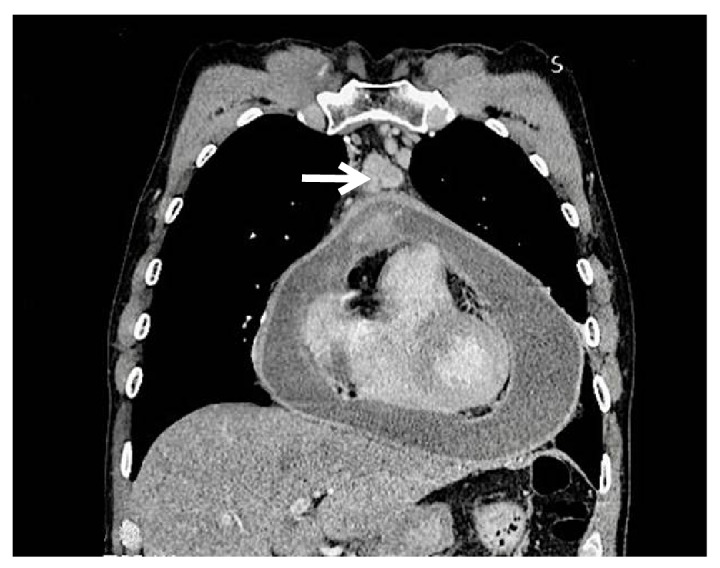
Contrasted chest computed tomography, mediastinal window. There is a 3-millimeter thickening and abnormal pericardial enhancement, pericardial effusion, which does not produce compression of the right ventricle, and pre-aortic adenopathy with heterogeneous enhancement (white arrow).

Chest X-rays showed global cardiomegaly with a rounded heart shape (figure 1). The chest tomography evidenced abundant homogeneous and hypodense pericardial effusion, thickening of the pericardial membrane, and enlarged lymph nodes (figure 2). An echocardiogram confirmed the accumulation of approximately 1,300 ml of pericardial effusion without hemodynamic compromise.

Taking into consideration the clinical and imaging characteristics, a pericardiocentesis was performed and 275 ml of yellowish liquid were obtained. The cytological analysis was negative for malignancy; the adenosine deaminase (ADA) measurement was 101 IU/l, the polymerase chain reaction for *Mycobacterium tuberculosis* (IS6110), smear microscopy, and culture (both MGIT and Lowenstein-Jensen) for mycobacteria were all negative.

**Figure 3:**
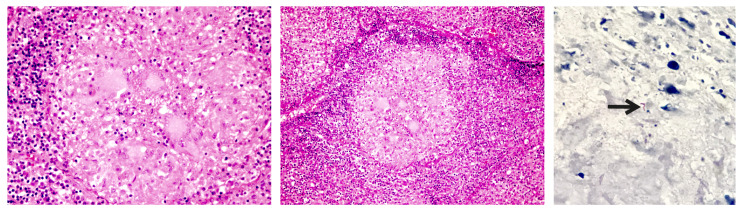
A and B. Pericardium compromised by chronic granulomatous inflammation with central necrosis and multinucleated giant cells. Hematoxylin-eosin. 40X. C. Zielh-Neelsen stain showing acid-fast bacilli (black arrow), 100X

Due to these inconclusive findings, biopsies of the pericardium and mediastinal lymph node were performed. The pathological examination showed an extensive chronic granulomatous reaction with necrosis and giant cells (figure 3) while the Ziehl-Neelsen staining showed acid-fast bacilli. Based on these results, anti-tuberculosis treatment plus prednisone was started. After the stabilization of his clinical condition, the patient was discharged and completed six months of anti-tuberculosis treatment with complete clinical recovery.

One to two percent of patients with pulmonary tuberculosis develops tuberculous pericarditis. However, it can also present as an isolated extrapulmonary form ^5^. In a Spanish series of 294 immunocompetent individuals with acute pericarditis, thirteen cases of tuberculous pericarditis were identified (4%), cardiac tamponade was observed in five cases, and constrictive pericarditis in six patients ^6^.

Pericardial involvement can occur by extension from the lungs, adjacent lymph nodes, the sternum or even the spine, as well as through hematogenous spread. Frequently, tuberculous pericarditis corresponds to the reactivation of a previous infection without an apparent primary site ^7^, which is probably what happened in the case we describe here.

Four pathological stages are described. Initially, there is a fibrinous exudate with polymorphonuclear infiltration and the formation of early granulomas. This is followed by a serosanguineous effusion with abundant lymphocytes and, finally, adsorption of the effusion with the onset of granulomatous necrosis, pericardial thickening, and fibrosis that can progress to constrictive pericarditis ^7^.

The clinical presentation is nonspecific and insidious, with symptoms such as fever, night sweats, and weight loss, usually preceding the cardiopulmonary symptoms, taking cough, dyspnea, and pleuritic pain being the most frequent ones ^8^. This was the case of our patient, who did not develop a hemodynamic compromise.

Regarding the diagnostic approach, this is established through the detection of *M. tuberculosis* bacilli in smear microscopy or culture of the pericardial fluid and/or the identification of bacilli or granulomatous inflammation in the pathological examination of the pericardium ^7^.

Pericardiocentesis is a common and useful procedure for the diagnosis of tuberculous pericarditis. The extracted fluid should be evaluated by smear and culture, ADA concentration, and cytology ^8^. In many cases, after this evaluation, the diagnosis is not reached and, therefore, a pericardium biopsy is necessary as described here.
